# Piezoelectric Micromachined Ultrasonic Transducers (PMUTs): Performance Metrics, Advancements, and Applications

**DOI:** 10.3390/s22239151

**Published:** 2022-11-25

**Authors:** Yumna Birjis, Siddharth Swaminathan, Haleh Nazemi, Gian Carlo Antony Raj, Pavithra Munirathinam, Aya Abu-Libdeh, Arezoo Emadi

**Affiliations:** Department of Electrical and Computer Engineering, University of Windsor, Windsor, ON N9B 3P4, Canada

**Keywords:** acoustic pressure, acoustic sensing, bandwidth, CMOS integration, electromechanical coupling, microelectromechanical systems, microfabrication, micromachined ultrasonic transducers, pulse-echo imaging, resonant frequency

## Abstract

With the development of technology, systems gravitate towards increasing in their complexity, miniaturization, and level of automation. Amongst these systems, ultrasonic devices have adhered to this trend of advancement. Ultrasonic systems require transducers to generate and sense ultrasonic signals. These transducers heavily impact the system’s performance. Advancements in microelectromechanical systems have led to the development of micromachined ultrasonic transducers (MUTs), which are utilized in miniaturized ultrasound systems. Piezoelectric micromachined ultrasonic transducers (PMUTs) exhibit higher capacitance and lower electrical impedance, which enhances the transducer’s sensitivity by minimizing the effect of parasitic capacitance and facilitating their integration with low-voltage electronics. PMUTs utilize high-yield batch microfabrication with the use of thin piezoelectric films. The deposition of thin piezoelectric material compatible with complementary metal-oxide semiconductors (CMOS) has opened novel avenues for the development of miniaturized compact systems with the same substrate for application and control electronics. PMUTs offer a wide variety of applications, including medical imaging, fingerprint sensing, range-finding, energy harvesting, and intrabody and underwater communication links. This paper reviews the current research and recent advancements on PMUTs and their applications. This paper investigates in detail the important transduction metrics and critical design parameters for high-performance PMUTs. Piezoelectric materials and microfabrication processes utilized to manufacture PMUTs are discussed. Promising PMUT applications and outlook on future advancements are presented.

## 1. Introduction

Ultrasonic transducers and sensors are known for their applications in non-destructive testing (NDT) and medical ultrasound imaging. In conventional bulk ultrasonic transducers, the fundamental resonant frequency is generated when the thickness of the piezoelectric layer is half of the wavelength. As such, conventional transducers have inherent frequency limitations, and high-frequency applications require very thin extensional piezoelectric layers [1,2], whereas in PMUTs, the resonant frequency does not solely depend upon the thickness of the piezoelectric layer, enabling better design flexibility for high-frequency applications.

Conventional transducers also have poor acoustic impedance matching with air or biological tissue mediums, limiting their bandwidth, resolution, and energy transfer to load media [1,3]. MUTs are membrane structures with better acoustic impedance matching with load medium, and therefore, they do not require the matching layer usually employed in conventional transducers to achieve broadband operation [2,4]. MUTs are of two types, based on their actuation principle: capacitive MUTs (CMUTs) and piezoelectric MUTs (PMUTs). Capacitive MUTs are electrostatically actuated and require high DC bias, limiting their easy integration with low-voltage electronics. For high performance, CMUTs require a small gap height for better sensitivity, which extends fabrication limitations. Unlike CMUTs, PMUTs are based on the piezoelectric effect, do not require a high DC bias voltage, and are not limited in deflection due to small gap height [5,6].

**Figure 1:**
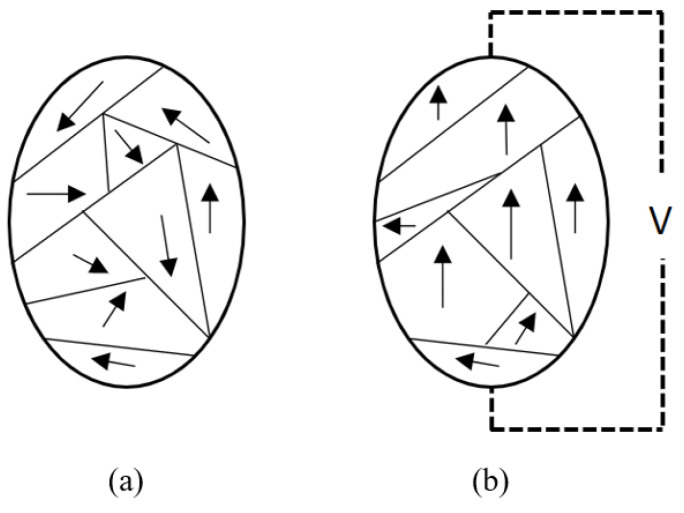
Schematic view of Electric dipole moments in (**a**) unpolarized piezoelectric material and (**b**) polarized piezoelectric material in the presence of an electric field.

Ultrasound is generated by different mechanisms, including the piezoelectric effect, magnetostriction, and the photoacoustic effect [3]. The most common of these mechanisms is the piezoelectric effect. Certain dielectric materials such as quartz crystals, semi-crystalline polyvinylidene polymers, polycrystalline, and piezoceramic exhibit piezoelectricity: a unique property that causes them to physically deform in the presence of an electric field or, conversely, to generate an electrical charge when mechanically deformed [7]. Piezoelectricity results from the spontaneous separation of charges in crystal structures. This phenomenon occurs by the displacement of positive ions with respect to negative ions within their crystal cells, which generate electric dipoles, as shown in Figure 1. When piezoelectric material is electrically polarized, it expands in the polar axis direction and compresses in the transverse direction. When a voltage is supplied to the poled piezoelectric material in the same direction as the poling voltage, it undergoes further expansion along the polar axis and contraction along the transverse direction, as specified by Poisson’s ratio. The piezoelectric material returns to its original pole dimensions in the absence of voltage. This phenomenon, which induces stress or strain in piezoelectric materials, is utilized to operate PMUTs [7,8].

A matrix relationship is utilized to express the piezoelectric effect and the mechanical, elastic stress–strain relationship simultaneously, as shown in Equations (1) and (2) [9,10,11].

(1) $$S=s^{E}T+d^{t}E$$

(2) $$D=dT+\varepsilon^{T}E\;$$

S and T represent longitudinal and shear components of stress and strain in the form of 1 × 6 column matrices, respectively, while *E* is the electric field, and $s^{E}$ is the compliance at constant electric field. A 3 × 6 matrix of the corresponding piezoelectric coefficients is represented by d and its transpose as $d^{t}$. $\varepsilon^{T}$ is a 3 × 3 matrix that contains permittivity coefficients when superscripts indicate which variable is held constant [11]. Amongst these coefficients, *d*_33_ of the piezoelectric coefficients links the applied voltage to expansion or contraction perpendicular to the piezoelectric layer. According to *d*_31_, the piezoelectric layer contracts laterally and deflects out of plane due to the applied voltage [11].

Piezoelectric constants $e_{31}$ and $d_{33}$ can be calculated by Equations (3) and (4), respectively, where *c* denotes the stiffness of the piezoelectric material. The first and second subscripts indicate the direction of the electric field or electric displacement and stress or strain, respectively [11,12].

(3) $$e_{31}=\frac{d_{31}}{s_{11}^{E}+s_{12}^{E}}\;\equiv \;e_{31}-\frac{c_{13}^{E}}{c_{33}^{E}}e_{33}\;\;\;\left|e_{31}\right|>\left|e_{33}\right|$$

(4) $$d_{33}=\frac{e_{33}}{c_{33}^{E}}\;\equiv \;d_{33}-\frac{2\;s_{13}^{E}}{s_{11}^{E}+s_{12}^{E}}\;\;\;\left|d_{31}\right|\;\langle \;|d_{33}|\;$$

The displacement field of the piezoelectric layer can be calculated using Equation (5)

(5) $$D_{3\;}=\varepsilon_{0}\varepsilon_{33}+e_{31}\left(S_{1}+S_{2}\right)+d_{33}\cdot T_{3}$$

where $\varepsilon_{0}$ represents permittivity and $\varepsilon_{33}$ is the dielectric constant of the piezoelectric material under in-plane stress, which can be calculated using Equation (6) [11].

(6) $$\varepsilon_{33}=\varepsilon_{33}^{T}-\frac{2d_{31}^{2}}{\varepsilon_{0}\;\left(s_{11}^{E}+s_{12}^{E}\right)}$$

## 2. PMUT Principle of Operation 

**Figure 2:**
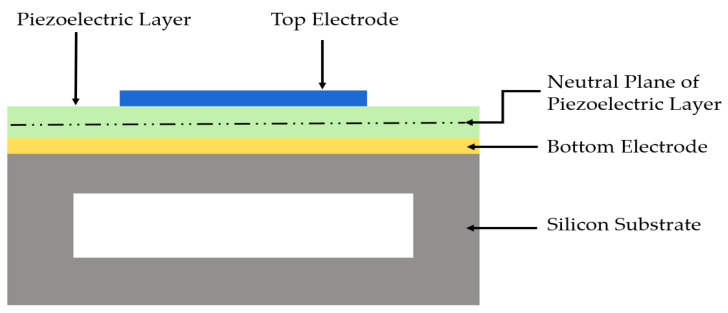
Schematic side view of a piezoelectric micromachined ultrasonic transducer (PMUT) cell.

**Figure 3:**
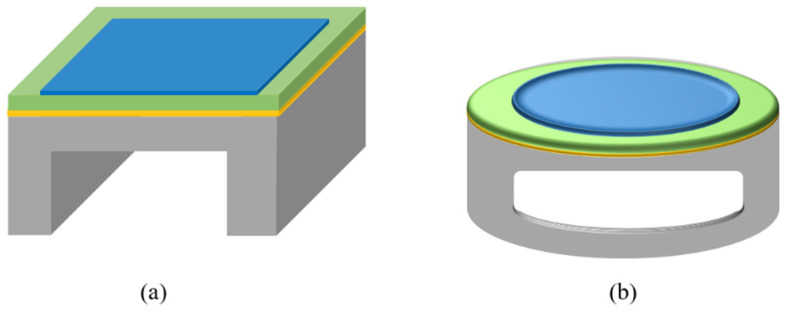
(**a**,**b**) Three-dimensional perspectives of PMUT cell.

PMUTs consist of a piezoelectric layer sandwiched between two electrodes on a supporting silicon substrate, as shown in Figure 2. Benefitting from the piezoelectric effect, the piezoelectric layer deforms when electrically excited using an AC voltage. This phenomenon creates vibration at the piezoelectric layer’s resonant frequency [5]. The deformation and bending of the piezoelectric membrane generate ultrasound waves. A three-dimensional view of PMUT cell is illustrated in Figure 3.

Unlike conventional piezoelectric transducers, PMUTs’ resonant frequency does not directly depend upon the thickness of the piezoelectric layer. In PMUTs, the resonant frequency is determined by the membrane shape, geometry, boundary conditions, and material properties [13]. For different geometric shapes of PMUT membrane (fully clamped), resonant frequency has been reported in previous works as given in Equations (7), (9) and (10).

For circular PMUT [13]:

(7) $$f_{C}=\frac{\alpha }{2\pi r^{2}}\sqrt{\frac{D}{\rho t}}$$

In Equation (7), r is the membrane radius, α is the first mode circular constant, D is the flexural rigidity, ρ is the membrane density, and t is the membrane thickness. The Flexural rigidity D is calculated using the Equation (8)

(8) $$D=\frac{E\;t^{3}\;}{12\pi (1-\upsilon^{2})}$$

In Equation (8), E is Young’s modulus, v is Poisson’s ratio, and t is the membrane thickness.

For a square PMUT [14]:

(9) $$f_{S}=\frac{\beta }{2\pi a^{2}}\sqrt{\frac{E}{(1-\upsilon^{2})\rho t}}$$

In Equation (9), a is the square side length, r is the membrane radius, β is the first mode square constant, E is Young’s modulus, v is Poisson’s ratio, t is the membrane thickness, and ρ is the membrane density.

For a rectangular PMUT [15,16]:

(10) $$f_{R}=\frac{\pi t}{4}\sqrt{\frac{E}{6\;\rho \left(1-\upsilon^{2}\right)}\;}\;\left[{\left(\frac{m}{a}\right)}^{2}+{\left(\frac{n}{b}\right)}^{2}\right]$$

In Equation (10), E is Young’s modulus, v is Poisson’s ratio, m and n are the membrane modes, ρ is the membrane density, t is the membrane thickness, and a,b are the length and width for the rectangle, respectively, where a≫b.

Electromechanical Coupling Coefficient (EMCC, $k^{2}$): The electromechanical coupling coefficient (EMCC) is defined as the total electrical energy applied converted into mechanical energy. EMCC is determined by the peaks of resonant and anti-resonant frequencies, i.e., $f_{r}$ and $f_{a}$, respectively, as given in Equation (11) [17,18].

(11) $$k^{2}=\frac{f_{a}{}^{2}-f_{r}{}^{2}}{f_{a}^{2}}$$

The EMCC is also determined by the change in capacitance and mechanical stiffness of the transducer membrane due to the piezoelectric actuation, as reported in [19].

(12) $$k^{2}=\frac{C_{m}}{C_{m}+C_{0}}$$

In Equation (12) above, $C_{m}$ is the motional capacitance when the transducer is in operation and $C_{0}$ is the static capacitance. The PMUT equivalent circuit model can further explain these parameters. 

## 3. PMUT Equivalent Circuit Model and Mechanical Modeling

**Figure 4:**
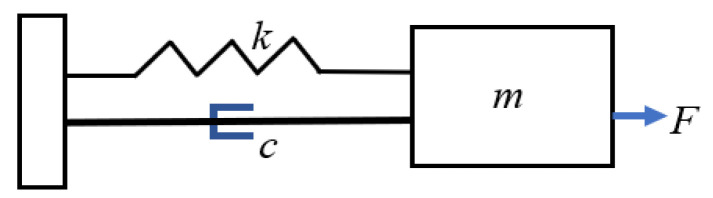
Mass–spring–damper system.

The equivalent circuit model implements lumped components to address the electrical and mechanical domains of the transducer in operation [18,20,21]. To represent the electrical domain, electrical impedance *Z* is defined by the impedance of the capacitor $C_{0}$. The capacitance $C_{0}$ is formed due to the piezoelectric layer sandwiched between two metallic electrodes. The mass–spring–damper system is used to represent the mechanical domain of the vibrating PMUT membrane, as illustrated in Figure 4 [21] where the piezoelectric layer acts as a mass connected to the spring. The mass m attached to the spring oscillates due to the external force *F*, the spring has the effective stiffness k, and c represents the damping coefficient due to the membrane deflection.

**Figure 5:**
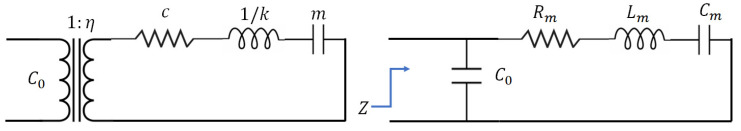
PMUT-equivalent circuit model.

The Van Dyke equivalent circuit representation, as shown in Figure 5, is used to model the vibrating PMUT membrane using an RLC circuit in parallel with a capacitance $C_{0}$ [20,21].

The circuit elements are determined as $R_{m}=\frac{c}{\eta^{2}}$, $L_{m}=\frac{m}{\eta^{2}}$, $C_{m}=\frac{\eta^{2}}{k}$, where $R_{m}$ represents the damping factor, $L_{m}$ represents the membrane mass, $C_{m}$ is the reciprocal of the membrane stiffness, and η is the electromechanical transduction.

## 4. Piezoelectric Materials and Fabrication Techniques for PMUT

The electromechanical performance and resonant frequency of PMUTs significantly depend upon the piezoelectric material properties. The piezoelectric materials commonly used in PMUTs include lead zirconate titanate (PZT) [5], polyvinylidene fluoride (PVDF) [22], aluminum nitride (AlN) [17,23], and zinc oxide (ZNO) [24]. Properties of commonly used piezoelectric materials in PMUT are listed in Table 1. PVDF is widely used in piezoelectric bulk transducers due to its chemical resistance and thermal properties [25]. One of the limitations of PVDF is that its piezoelectric properties degrade during fabrication if exposed to high temperatures for longer periods. Moreover, PVDF is not preferred for MEMS applications due to difficult thin film production and poor adhesion to substrates and metal, which requires a bond between film and wafer [25]. PZT thin films are the most widely used ferroelectric materials, with higher piezoelectric coefficient between −8 and 12 C/m^2^ [26]. The deposition of thin PZT films can be done through a variety of methods, including sputtering, sol-gel, and metal organic chemical vapor deposition (MOCVD) [27]. The sol-gel method is used for uniform deposition at low cost. PZT-based PMUTs, including niobium-doped PZT (PZNT) PMUTs with high piezoelectric coefficients (−23 C/m^2^), are used in applications utilizing a low input voltage while attaining high sensitivity [28,29]. Thin PZT ceramic films have also been reported to be used in PMUTs for photoacoustic applications due to their high piezoelectric coefficients [30,31]. Monocrystalline PZT has a high piezoelectric coefficient (14 C/m^2^) and smaller dielectric constant compared to polycrystalline PZT films and has a higher curie temperature [32]. AlN and ZnO are also commonly used piezoelectric materials which are non-ferroelectric materials, and their deposition is controlled to provide the alignment of polarization direction, whereas the ferroelectric PZT can be poled after the deposition of the film [33,34]. The piezoelectric coefficient of AlN is very similar to ZnO, but ZnO has certain limitations, including high dielectric loss at low frequencies and fast diffusion of Zinc ions, resulting in issues with IC integration [17,33]. High temperatures are required for thin film deposition of PZT through sol-gel, chemical deposition, and sputtering. High-temperature thin film PZT deposition imposes difficulties on MEMS integration with CMOS due to temperature limitations during fabrication for the CMOS process [17,35]. Another limitation of PZT-based PMUTs is the release of harmful lead into the atmosphere during high-temperature deposition [36,37]. Alternatively, AlN thin films are deposited through low-temperature sputtering, thereby facilitating CMOS compatibility and enabling monolithic integration [17]. Due to its moderate piezoelectric coefficient, AlN can be utilized for transducer applications in both sensing and actuation [29].

Based on the approach utilized to define the PMUT diaphragm, PMUT fabrication techniques include back-side etching, front-side etching, and sacrificial release [13].

### 4.1. Back-Side Etching

**Figure 6:**
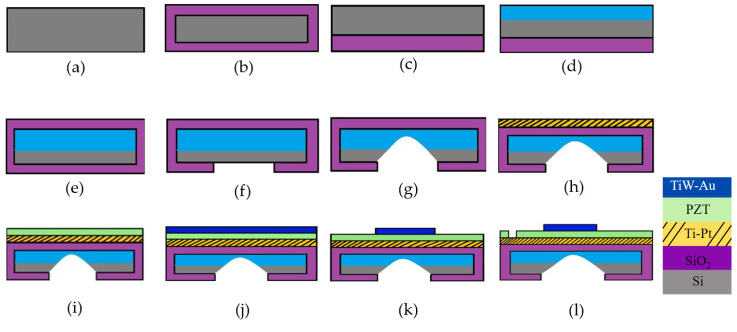
PMUT fabrication using the back-side etching process. (**a**) (100) Si wafer as a substrate, (**b**) Wet oxidation, (**c**) SiO_2_ etching using BOE, (**d**) Boron diffusion, (**e**) Formation of sacrificial layer by LTO, (**f**) Oxide etch using BOE, (**g**) Si etching by EDP, (**h**) Bottom electrode Ti–Pt deposition by e-beam evaporation, (**i**) PZT deposition by sol-gel technique, (**j**) Top electrode TiW–Au deposition by sputtering, (**k**) Top electrode etching using KI_3_ and H_2_, (**l**) PZT etching using HCl:HF:H_2_O.

Back-side etching is one of the alternative procedures to define the diaphragm in PMUT fabrication. This etching procedure starts with a realization of 500 nm thick SiO_2_ insulation layer on the silicon substrate by wet oxidation at 1050 °C, as illustrated in Figure 6a,b. The SiO_2_ layer is then partly etched using buffered oxide etchant (BOE), a solution of HF, NH_4_F, and H_2_O, as shown in Figure 6c. The membranes are fabricated by anisotropic silicon wet etching with boron-doped silicon as an etch stop (Figure 6d). Borosilicate glass formed at the wafer surface due to boron diffusion is removed using BOE. It is to be noted that the diffusion rate of boron controls junction depth. The low-temperature oxidation method (LTO) is used to grow the sacrificial oxide layer and then etched using BOE to avoid impurities at the boron surface. This ensures a clean surface for the final oxide layer, as shown in Figure 6e,f. The final oxide layer is used to provide silicon windows or etch masks through ethylene diamine and pyrocatechol (EDP) etching. The etch window of the back-side is further patterned using photolithography, and the silicon wafer is etched using EDP etchant, as given in Figure 6g. Other etchants such as potassium hydroxide (KOH) [40], which forms 54.7° sloping walls [2], and tetramethylammonium hydroxide (TMAH) [13] are widely used. To form perpendicularly defined sidewalls [13], deep reactive ion etching is commonly used [41,42].

The piezoelectric active layer, namely PZT (lead zirconate titanate), is deposited as a thin layer using the sol-gel method [13]. The bottom side electrode, consisting of a 200 nm platinum (Pt) electrode and 10 nm of titanium, is deposited by e-beam evaporation on the oxide layer, as depicted in Figure 6h. The sputtering technique is used to deposit the top electrode made of 10 nm of titanium tungsten (TiW) and 200 nm of gold (Au). Photolithography is then used to pattern the top electrode. The patterning of the PZT layer is done using HCl:HF:H_2_O etchant to get access to the bottom electrode (Figure 6i–l).

### 4.2. Front-Side Etching

**Figure 7:**
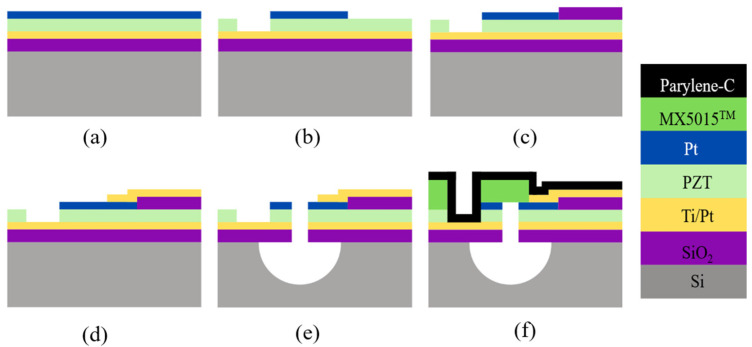
PMUT fabrication procedure for defining diaphragm via front-side etching technique. (**a**) RF sputter deposition applies a thick coat of PZT and a Pt electrode top-layer on a silicon wafer layered with SiO_2_ and Ti/Pt. (**b**) Reactive ion etching (RIE) exposes the Ti/Pt electrode and patterns the Pt top-layer along with contact lithography. (**c**) A layer of SiO_2_ is applied as an insulation pad via sputter deposition and lift-off to prepare for the fabrication of the electrode fan-out. (**d**) A conformal connection is formed using sputtered and patterned Ti/Pt. (**e**) Access to the bottom Si layer diaphragm is created by means of an etch via through the Pt, PZT, Ti/Pt, and SiO_2_ stack. (**f**) The diaphragm is then laminated with negative photoresist film (MX5015) and coated with parylene to seal the vias.

Another route to define the diaphragm cavity is carried out by etching the Si substrate through the membrane from the front surface [43,44]. Figure 7 shows the schematic of fabrication process flow for PMUTs with the release of circular diaphragms from the front-side. In this case, the Si substrate is isotropically etched through a predefined etch via hole. The final configuration of the fabricated membrane of PMUTs is to consist of a SiO_2_/Ti/Pt/PZT/Pt polymer stack.

A 1 µm thick layer of PZT is deposited over a platinized Si wafer through RF sputtering at room temperature. The wafer has a 1 µm thick SiO_2_ layer, 20 nm of Ti, and 50 µm of (111)-oriented Pt that is cleaned thermally before commencing the deposition. To improve film density and prevent film cracking, the film is deposited in three successive layers of thicknesses 500 nm, 250 nm, and 250 nm, which are annealed in the oxygen atmosphere after each deposition, as shown in Figure 7. The top electrode in the form of a Pt layer is deposited on the PZT layer via sputtering and annealed at 500 °C for good adhesion. The top electrode is then patterned using contact lithography and reactive ion etching (RIE) processes. To access the bottom Ti/Pt electrode, RIE of the exposed PZT is done with a thick photoresist mask. It should be noted that the bottom Ti/Pt electrode is sputtered through lift-off to ensure conformal connection to the top electrode. The etch via is made to access the bare silicon below the stack to ensure the front-side release of the diaphragms. The stack of Pt, PZT, Ti/Pt, and SiO_2_ is etched using the RIE process. To improve acoustic performance and seal the vias, the diaphragms are laminated with a 15 µm thick negative photoresist film (MA5015). Before starting the lamination process, the dies are cleaned in oxygen plasma for improved lamination. The laminate is subsequently patterned to expose bond pads for wire bonding, and the package is then cleaned in an Ar plasma and coated with parylene. The surface is activated in oxygen plasma to ensure the hydrophilicity of the parylene. The main advantage of front-side release is the ease of creating cavities of smaller diameters. 

### 4.3. Sacrificial Layer Release

Sacrificial layer formation and release are essential process steps routinely used in the microfabrication of PMUTs [45]. PMUT fabrication starts with the formation of a sacrificial layer on the Si substrate. After fabrication completion and patterning of all the layers of the diaphragm structure, the sacrificial layer is removed through a small opening, resulting in a cavity forming below the diaphragm. The first step in PMUT fabrication is the formation of a phosphorus-doped low-temperature oxide layer as a sacrificial layer on the Si substrate, followed by a thin silicon nitride (Si_3_N_4_) layer using the low-pressure chemical vapor deposition (LPCVD) technique. The e-beam evaporation technique is used to deposit the Ti/Au bottom electrode layer. 8 µm diameter access holes are made for realizing desired final diaphragms through wet etching of the bottom electrode layer and plasma etching of the silicon nitride layer. After completing the patterning of the bottom electrode layer, a thin ZnO layer of thickness 0.3 µm is deposited using the sputtering technique and functions as the piezoelectric layer. The ZnO layer is further patterned by wet etching into ring shapes of 30 µm and 80 µm as the inner and outer diameters, respectively. The top electrode consisting of Cu and Au is deposited using the e-beam evaporation technique and patterned into ring shapes through a lift-off process. In the last step, the wet etching process is used to release the sacrificial layer.

## 5. PMUT Performance Metrics and Critical Design Parameters

Transduction metrics and the associated critical design parameters are investigated to evaluate transducer performance. PMUTs offer several advantages in comparison to conventional bulk transducers due to easy and cost-effective microfabrication and the development of 2D arrays [13,46]. PMUTs do not require high DC bias voltage, as in the case of CMUTs, which is desirable for CMOS compatibility and applications. However, PMUTs have limitations with respect to electromechanical coupling and bandwidth [47,48]. Moreover, at higher frequencies, the volumetric displacement of the transducer membrane is reduced, and therefore, the acoustic pressure output of the PMUT is limited [49,50]. To develop high-performance PMUTs, current research focuses on enhancing the electromechanical coupling coefficient, bandwidth, and acoustic output pressure.

### 5.1. Electromechanical Coupling Coefficient (EMCC)

In previous works, comprehensive modelling of PMUTs using equivalent circuits has been developed for analytical equations of the EMCC [18,19,51]. These analytical models demonstrate that a high EMCC is dependent on material properties and optimization of geometric parameters, including electrode topology, electrode coverage, and layer thickness optimizations [5,41,51]. PMUT design parameters, namely the electrode coverage, materials, and thickness, have been studied in previous works to obtain a higher electromechanical coupling coefficient [5,51]. The development of piezoelectric material with a high piezoelectric coefficient has also been reported to improve EMCC in PMUTs [52,53].

Muralt et al. [5,41] developed a PMUT model to enhance electromechanical coupling by optimizing electrode coverage and silicon layer thickness. The PMUT membrane was fully clamped with a 270 µm diameter, 2 µm thick lead zirconate titanate (PZT) film and a silicon layer thickness of 4.5 µm. The coupling performance of the PMUT was optimized for an electrode coverage of 65% of the membrane radius and a silicon layer thickness of 20% greater than the piezoelectric layer thickness. The PMUT reported an electromechanical coupling coefficient of 6%. However, the electrode coverage and layer thickness optimizations were predicted based on predetermined piezoelectric thin film material properties. Hence, Muralt et al. concluded that material properties are the most significant factors that determine the electromechanical performance of the PMUT, but electrode coverage, relative layer thickness, and diaphragm structures contribute to the performance as well.

An analytical equivalent circuit model for PMUTs has been proposed by Smyth et al. [51] for electrode size optimization to improve electromechanical coupling in PMUTs. The optimal design reported 60% of electrode coverage with respect to membrane radius. The analytical modeling with lumped circuit parameters reported in [51] serves to quantify transducer performance metrics including EMCC, bandwidth, and output pressure in future works.

Amar et al. [21] reported an optimized design and circuit model of PMUT using lumped element parameters. The diameter of the designed PMUT was 100 µm, with piezoelectric layer and silicon layer thicknesses of 2 µm and 4 µm, respectively. In the optimized model, EMCC was found to be maximized with an electrode coverage of 53% of the membrane radius and a silicon thickness 17% larger than the piezoelectric film thickness [21]. The reported PMUT used PZT as the piezoelectric layer and obtained an EMCC of 4.2%.

**Figure 8:**
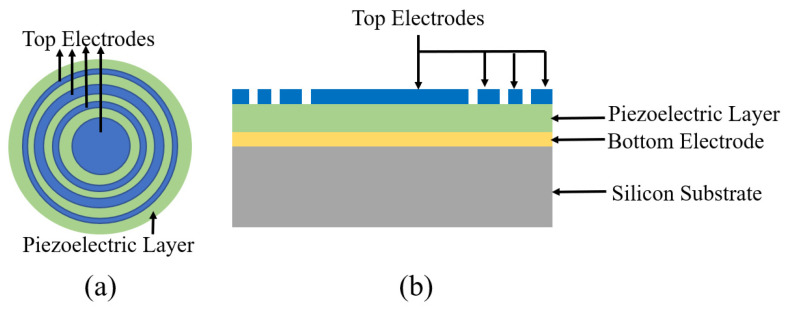
Multiple-electrode PMUT. (**a**) Top view of four-electrode PMUT, (**b**) Cross section side view of four-electrode PMUT.

Modelling and design of PMUTs with multiple electrodes was reported by Sammoura et al. [54]. The reported PMUT was modelled with a four-electrode design, as illustrated in Figure 8, with a 2 µm thick PZT as the piezoelectric layer and a 135 µm membrane radius. The EMCC reported for the four-electrode PMUT was 13.72%, which is 211% higher than the state-of-the-art single-electrode PMUTs.

**Figure 9:**
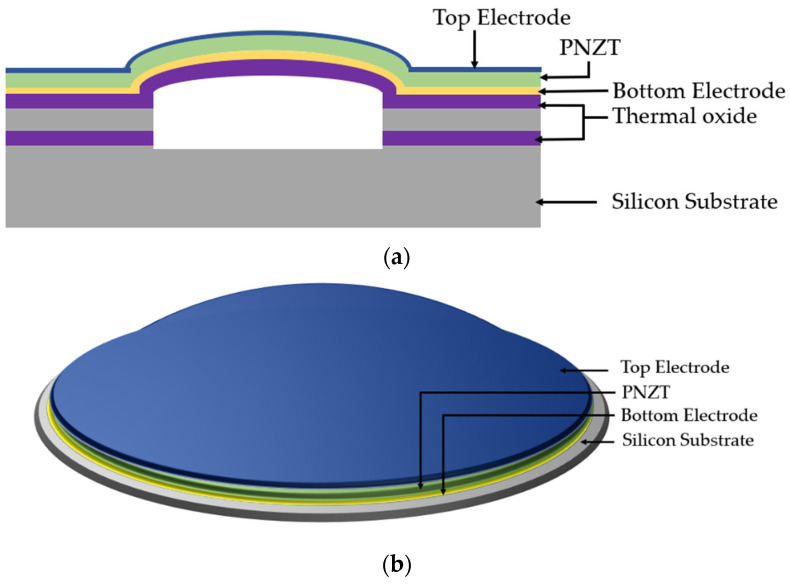
(**a**) Cross-sectional side view of dome-shaped PMUT; (**b**) 3D view of dome-shaped PMUT.

Three-dimensionally structured membranes, such as the curved [55] and dome-shaped [56,57], have been reported in previous research to enhance the EMCC of PMUTs. In 3D curved structures, vertical deformation of the piezoelectric layer is generated, resulting in a higher EMCC [58]. A dome-shaped PMUT, as illustrated in Figure 9 and reported by Hajati et al. [57], consists of niobium (Nb)-doped PZT (PNZT) as the piezoelectric material. The niobium dopant increases the piezoelectric coefficient by 70%, resulting in a better EMCC. The piezoelectric layer is in the form of a three-dimensional dome sandwiched between thin film top and bottom electrodes. The dome-shaped membrane with a cavity diameter of 75 µm eliminates the structural silicon layer, as shown in Figure 9, which induces a stretching mode compared to the conventional bending mode in planar PMUTs. The stretching mode offers a better EMCC and acoustic sensitivity [57]. The dome-shaped PMUT reported an EMCC of 45%, which is higher than the state-of-the-art PZT-based PMUTs.

**Figure 10:**
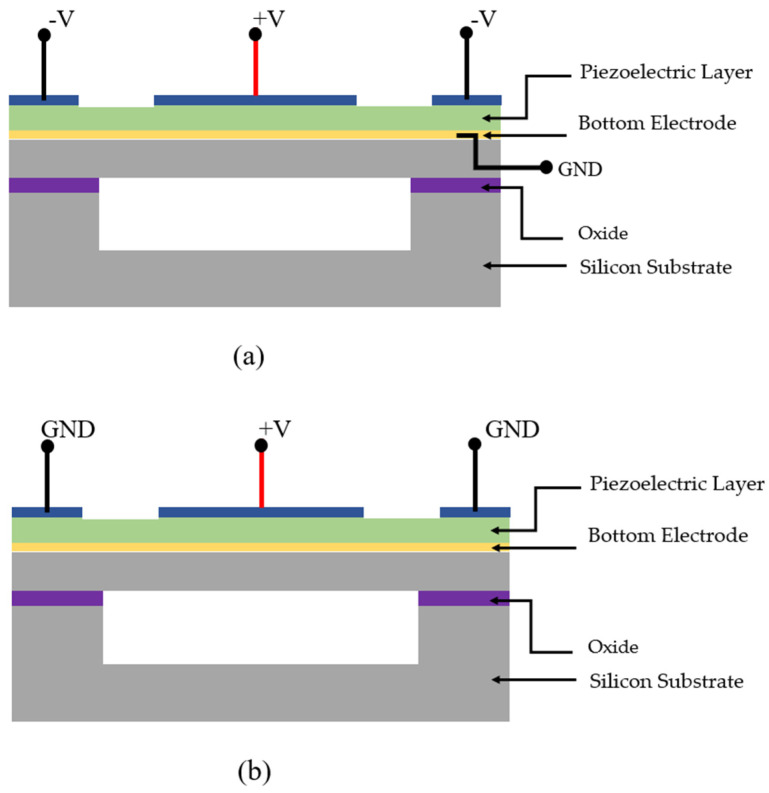
(**a**) PMUT with differential transduction; (**b**) PMUT with series transduction.

As an alternate technique to modelling device structural parameters and electrode topologies, the driving mechanism of PMUTs has also been demonstrated to improve the coupling coefficient [51,59,60]. A PMUT with differential transduction consists of two top electrodes, a center and surrounding ring electrode, and a common bottom ground electrode as shown in Figure 10a. But the differentially driven PMUT requires a minimum of three electrical contacts, leading to complex interconnects and pads for larger PMUT arrays [59]. In the series transduction PMUT, the two top electrodes are driven such that voltage is applied to the center electrode and the ring electrode is grounded, forming two piezoelectric transducers in series, as shown in Figure 10b. The floating bottom electrode will attain half of the voltage, and only half of the voltage is applied between the each of the top electrodes and bottom electrode. The electrical capacitance of the transducer is also halved, resulting in a doubled EMCC as compared to a traditional single-electrode-driven PMUT.

Several approaches have been reported to obtain a high EMCC, but the coupling coefficient of PMUTs is still limited. Hence, further research on PMUTs is focused on broad bandwidths to achieve a better energy conversion efficiency and performance of PMUTs.

### 5.2. Bandwidth

Wide-band ultrasonic transducers can generate acoustic pulses of shorter lengths. A shorter pulse length corresponds to a better resolution, known as the axial imaging resolution [3]. The axial resolution is the ability of the imaging transducer to differentiate closely spaced structures [3]. One way to achieve a shorter pulse length is through higher frequencies of operation. However, higher frequencies have a higher rate of attenuation, which limits the detection range of imaging. To obtain a reasonable detection range while achieving a shorter pulse length for better resolution of ultrasonic imaging, the frequency bandwidth of the transducer is enhanced.

Conventional bulk transducers have inherently poor bandwidth due to the large acoustic impedance mismatch between the transducer and human body tissue [1]. MUTs, due to their membrane structure, have better acoustic impedance matching with body tissue. CMUTs offer high bandwidth; however, this high bandwidth performance of CMUTs requires a large operating voltage which is not desirable in medical imaging applications [13]. Hence, PMUTs are considered a viable option for wide-band ultrasonic imaging. Though analytical modelling of PMUTs suggests their potential to achieve 100% bandwidth [2], experimentally, PMUTs are still limited in bandwidth due to the residual stress induced by the fabrication process [6,41]. Several approaches have been reported to overcome the bandwidth limitations of PMUTs.

Hajati e al. reported on a PMUT array with 57 dome-shaped membrane cells with cavity sizes ranging from 74 µm to 90 µm in diameter and an interelement pitch of 400 µm. Each PMUT cell generated distinct resonant peaks. When the cells of the PMUT array were actuated together in liquid, the resonant peaks were merged, resulting in a wider bandwidth [57]. The achieved bandwidth from the PMUT array was 55%. A complete array of PMUT cells causes this implementation of wide bandwidth instead of single pixel. Hence, there is a need for a PMUT design for single-pixel wide-band imaging to produce ultrasound images with improved resolution without mechanically scanning.

**Figure 11:**
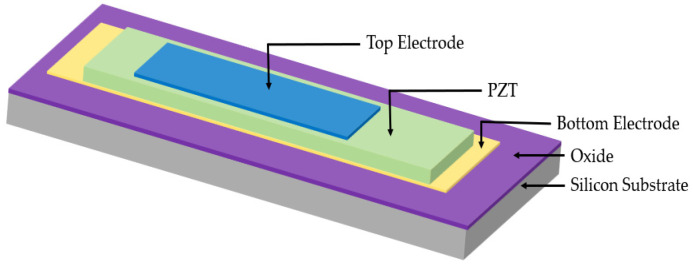
Rectangular PMUT with a large aspect ratio (mode-merging PMUT).

Single PMUT design with broader bandwidth, as reported by Wang et al. [61], consists of a rectangular ribbon-shaped membrane with a large aspect ratio, i.e., a considerable length-to-width ratio (L/W), as illustrated in Figure 11. The rectangular PMUT reported contains membrane dimensions of 1550 µm × 250 µm and an aspect ratio of 6.2 which generates distinct, closely spaced resonant mode peaks. These resonant peaks are merged when the PMUT is operated in a liquid or a damped acoustic medium. The mode-merging effect results in a wider bandwidth. The rectangular PMUT reported a fractional bandwidth of 94% when driven by a 20 V electric pulse.

Another mode-merging technique for the broadband operation of PMUTs has been reported by Eovino et al. [62]. In this technique, a ring-shaped PMUT, when operated in a liquid medium, generates two resonant peaks; one is the primary resonance which is independent of the acoustic load medium, as in the case of air-coupled PMUTs; the other resonant peak is due to the acoustic interactions with the load medium, which occurs due to the ring-shaped structure of the PMUT. This ring-shaped PMUT reported a bandwidth 60% higher than the bandwidth of previously reported PMUTs. The mode-merging and mode-overlapping methods have significantly improved the bandwidth of PMUTs. However, the mode-merging effect is highly sensitive to acoustic load medium density [62].

**Figure 12:**
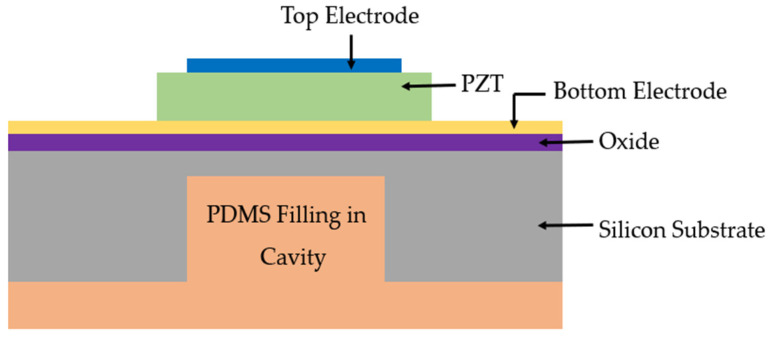
Three-dimensional cross-sectional side view of PMUT with backing layer.

An alternative technique to improve the bandwidth of PMUTs was demonstrated by Wang et al. [63]. The proposed PMUT implements a backing layer through deep silicon etching and polydimethylsiloxane (PDMS) back-filling into the etched cavity, which is formed by the release of the device structural layer through deep reactive ion etching (DRIE), as shown in Figure 12 [63]. To evaluate the bandwidth performance, an impulse response test was performed in water with a 30 V input pulse on the PMUT with backing layer. The PMUT with the backing layer was reported to double the bandwidth compared to PMUTs without the backing layer.

### 5.3. Acoustic Pressure Output

Higher acoustic pressure output is required to achieve larger imaging depth and detection ranges for medical imaging and range-finding applications. The acoustic pressure output can be related to the axial pressure amplitude *P*(*z*) at a distance *z* from the transducer, given by Equation (13) [35,64].

(13) $$P\left(z\right)=\frac{\;P_{0}\;R_{0}}{z}=\frac{2\pi d_{0}f_{0}{}^{2}\rho S\;}{z}$$

In Equation (13) above, $\;R_{0}\;$ is Rayleigh distance ($\frac{S}{\lambda })$ (where λ is the wavelength of the acoustic signal);$\;P_{0}$ is the pressure at surface S of the transducer given by $\;P_{0}=u_{0\;}Z_{0}$ (where $u_{0}$ is the membrane velocity and $Z_{0}$ is the acoustic impedance of the media). $Z_{0}$ is given by $Z_{0}=\rho c$, where ρ is the mass density of the acoustic medium and c is the speed of sound. $u_{0}$ is given by $u_{0}=2\pi d_{0}f_{0}$, where $d_{0}$ is the membrane displacement and $f_{0}$ is the resonant frequency. According to Equation (13), acoustic pressure output scales with membrane displacement. For high acoustic pressure transfer, the PMUT membrane should exhibit large displacement [64].

Conventionally, PMUT cells have fully clamped boundaries [5,41]. A decrease in the clamped boundary of the PMUT membrane increases the volumetric displacement, resulting in higher acoustic pressure. Wang et al. [65] developed a partially clamped PMUT using an isolation trench cavity. The reported PMUT had a diameter of 96 µm with an isolation trench of 10 µm width and 6 µm depth. The average membrane displacement was reported to improve by 61%, with a 76% improvement in acoustic output compared to fully clamped PMUTs. The use of an isolation trench at the edge of the PMUT membrane reduces the tensile stress caused by the deflection of the membrane.

Liang et al. developed a ‘pinned’-boundary PMUT [66] in which the pinned structure is obtained by etching the piezoelectric layer. The electrode coverage is realized at 80% of the membrane radius, which is defined by the back-side etching. A comparative study of clamped and pinned-boundary PMUT structures was undertaken using the same diaphragm radius of 91 µm. The pinned-boundary PMUT exhibited a 983 nm/V peak displacement at resonance, and the clamped PMUT showed a 368 nm/V peak displacement at the same resonant frequency. The pinned PMUT showed a 220% improvement in displacement per volt for the same radius as the clamped PMUT. For the pinned-boundary PMUT, the pressure output at a 2 mm distance was stated to be 38 Pa/V compared to the pressure output of the clamped PMUT of 11 Pa/V, which translates to a 227% improvement.

**Figure 13:**
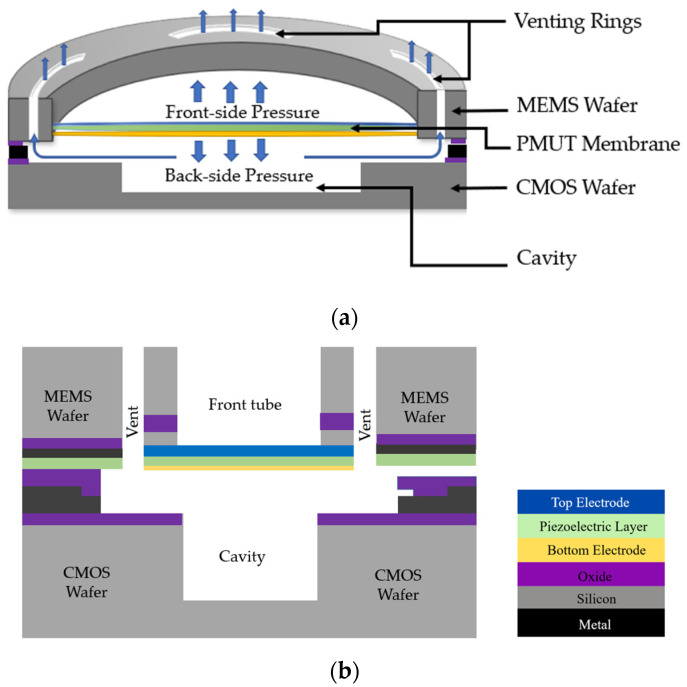
(**a**) Three-dimensional cross-sectional view of PMUT with venting rings. (**b**) Cross-sectional view of PMUT with venting rings with MEMS bond on CMOS.

In conventional PMUTs, the acoustic pressure emitted from the back-side of the membrane is not utilized and wasted. Rozen et al. [67] reported a novel PMUT structure for obtaining high acoustic pressure output. The reported PMUT comprises a circular structure with a front tube, back cavity, and ring-shaped acoustic venting holes, as shown in Figure 13a. The tube length for the PMUT reported was set at 210 µm with venting rings of a depth of 2.5 µm, diaphragm radius of 182 µm, and cavity height of 50 µm. The piezoelectric layer used in this PMUT design is aluminum nitride (AlN) with a thickness of 1 µm, and the operating frequency of the PMUT is 760 kHz. Through the venting rings, the back-side acoustic pressure is recycled and redirected to the front-side, increasing the overall acoustic pressure output of the PMUT. The reported PMUT experienced an increase in acoustic pressure output by 4.5 dB compared to conventional PMUTs without venting rings. Inefficient back cavity design can further limit the acoustic output due to damping induced by squeezed films; therefore, the radius of the venting rings needs to be optimized to maximize acoustic pressure output [67]. The proposed PMUT with venting rings was fabricated with PMUT on a CMOS wafer through wafer-level bonding. For efficient back cavity design and to mitigate squeezed film damping, the CMOS wafer included a DRIE etch to implement a 50 µm cavity, as shown in Figure 13b.

**Figure 14:**
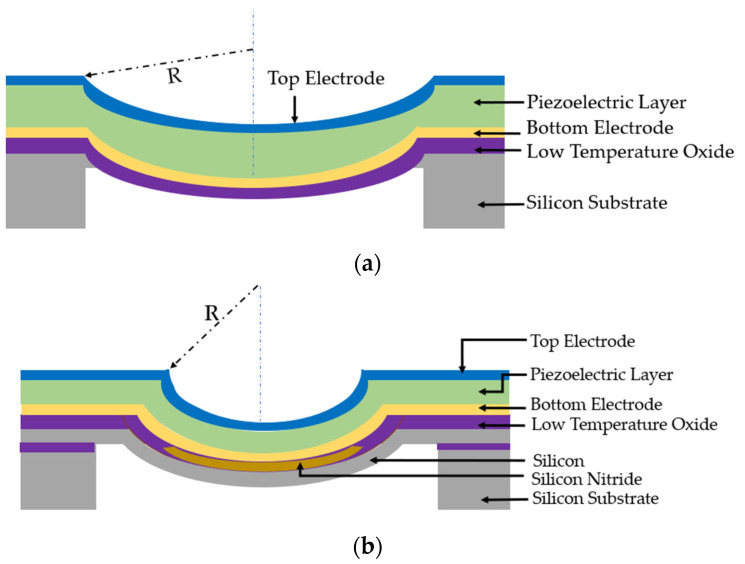
(**a**) Cross-sectional view of curved PMUT with concave diaphragm. (**b**) Cross-sectional view of stress engineered self-curved PMUT with concave diaphragm.

In planar PMUTs, a structural silicon layer is required to ensure non-zero piezoelectric moment along the neural axis of the PMUT structure [58]. In curved PMUTs, the in-plane stress is converted to a vertical mechanical component, which results in a better EMCC and acoustic pressure [58,68]. In curved PMUTs, the piezoelectric layer can serve as the structural layer, so they do not need an extra silicon layer as in planar PMUTs [58]. Curved PMUTs consist of a concave diaphragm with a radius of curvature R formed by silicon wet-etching process, as illustrated in Figure 14a. A curved AlN-based PMUT was developed by Akbari et al. [55] which reported a center displacement of 45 nm/V at resonance, which is 50 times higher than other AlN-based PMUTs. However, the curved structures with the desired radius of curvature render a large portion of the PMUT inactive, reducing the fill factor (transducer active area). Acoustic efficiency of PMUTs increases when the fill factor of the design is maximized [69]. Akbari et al. [70] further developed a residual stress-engineered self-curved PMUT to achieve controllable designs for diaphragm of curvature. The stress-engineered design consists of a thin silicon nitride (SiN) layer with a residual tensile stress of 650 MPa on the inner portion surrounded by 180 MPa of compressive stress by low-temperature oxide, as shown in Figure 14b. After the release of the stresses, the curved structure of the PMUT diaphragm is self-formed. The radial coverage of the SiN affects the center deflection [70]. The curved PMUT with 200 µm radius and 2 um AlN thickness and 50% of SiN coverage reported a center deflection of 2.7 µm and displacement sensitivity of 40 nm/V.

When designing PMUT arrays, a large active area (fill factor) is desirable for high acoustic pressure. Hence, to increase acoustic efficiency of PMUT, the fill factor of the array needs to be maximized [60]. To achieve a high fill factor, the interelement spacing and pitch of the PMUT array needs to be optimized. Previous PMUT arrays have been fabricated with wet isotropic etching that produces larger PMUT dimensions and pitches, resulting in a low fill factor [2]. Alternatively, wafer deep reactive ion etching (DRIE) is used to produce PMUT arrays with diameters of 65 µm and pitch of 100 µm [42,71]. However, diameter variations due to the DRIE process affect the center frequency of PMUTs. The cavity silicon-on-insulator (SOI) process is utilized to implement precise cavity dimensions in PMUTs, resulting in 4 times the fill factor than DRIE processes [60]. The cavity SOI process facilitates the fabrication of PMUTs with smaller diameters and fine pitches to achieve a high fill factor [60].

**Figure 15:**
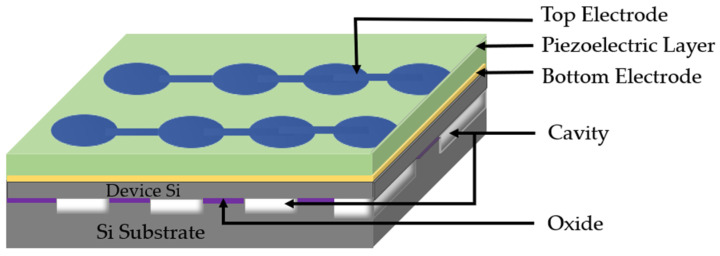
Three-dimensional schematic of a PMUT array based on cavity SOI.

A 9 × 9 PZT-based PMUT array with a 40 µm membrane diameter fabricated with the cavity SOI process has been reported by Lu et al. [60], as illustrated in Figure 15. PZT was used for piezoelectric layer of the PMUTs. The acoustic pressure output of the array was measured with a needle hydrophone which reported a 58 kPa peak-to-peak pressure amplitude at a distance of 1.2 mm in liquid Flourinert. To achieve a focused, higher-pressure output, electronic beam-forming technique is utilized, where PMUTs in the array are activated with phase shift delays. A 15 × 9 PMUT array with a 40 µm element diameter and 60 µm pitch was driven with 18 V_pp_ at 10 MHz, resulting in 450 kPa peak-to-peak pressure output.

**Figure 16:**
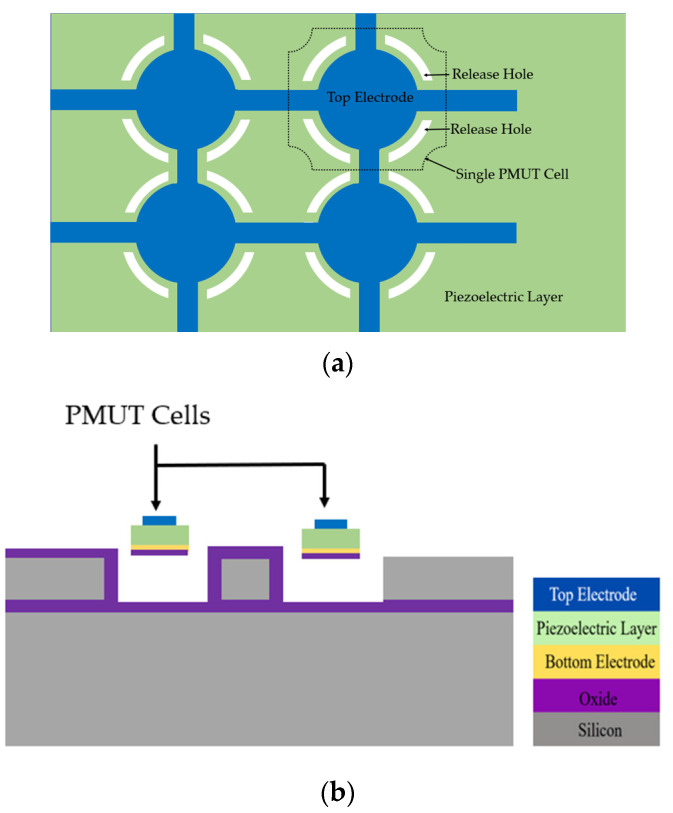
(**a**) Top view of PMUT cells with release holes on the edges, (**b**) cross-sectional view of fabricated PMUT cells with SOI substrate.

A systematic design optimization was implemented by Smyth et al. to achieve improvement in bandwidth and coupling while maintaining high acoustic output pressure [47]. In this systematic design approach, electrode coverage and piezoelectric layer thickness are optimized for high coupling. Then, multi-cell array patterning and spacing are selected to achieve a high fill factor for high acoustic efficiency. Fill factor is increased by utilizing release holes along the edges of the PMUT cells, as shown in Figure 16. These release holes along the cell edge further increase the coupling by relaxing the boundary walls. The fabricated PMUT arrays demonstrated a large packing density and a high coupling coefficient of 9% for each PMUT cell through frequency-based measurement.

**Figure 17:**
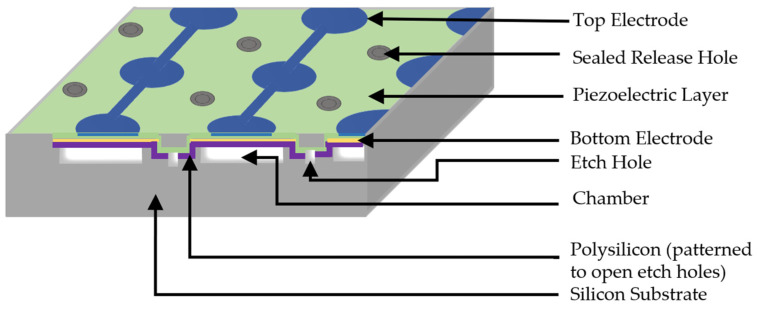
Three-dimensional cross-sectional view of PMUT array illustrating structure details and magnified view of etch holes.

A 50 × 50 PMUT array developed with elements 48 µm in diameter and with 52 µm pitch has been demonstrated by Jiao et al. [72]. The array elements are connected in rows and columns through top and bottom electrodes as illustrated in Figure 17. The PMUT arrays are designed with surrounding etch holes through micro etching channels such that each etch hole is shared by surrounding PMUTs. This design strategy of reusable etching holes decreases interelement spacing, thereby improving the fill factor of the PMUT arrays. The PMUT elements are built with AlN as the piezoelectric layer. The AlN is used due to its lower dielectric constant for better receive sensitivity and low-temperature deposition compatible with CMOS processes [17]. A fill factor of 67% was obtained for the PMUT array with reusable etch holes, which is approximately two times larger than the previously reported PMUTs [42,71,73]. A peak displacement of 3.25 nm/V was reported when a single PMUT was driven at its center frequency of 12.62 MHz with an 8 V sinusoidal signal. The electromechanical coupling coefficient of the PMUT was calculated at 1.6% from impedance measurements.

Creative multifrequency approaches have been developed in recent works on PMUTs to achieve high performance where the trade-off between acoustic pressure and bandwidth is addressed. In ultrasonic imaging applications, the frequency and bandwidth determine the imaging resolution. However, higher frequencies have a greater rate of attenuation. Conventional PMUTs operate at a single frequency, which imposes a trade-off between resolution and detection range for different applications [74]. A multifrequency PMUT proposed by Wang et al. [74] offers the ability to electrically switch between three different frequencies, including 2 MHz, 3.19 MHz, and 5.84 MHz. The frequency switching is achieved by activating different top electrode sets of the PMUT. The proposed multifrequency PMUT consists of 400 µm × 170 µm rectangular cavities built by cavity SOI. The piezoelectric layer used is AlN of thickness 1 µm with molybdenum (Mo) top and bottom electrodes, each of thickness 0.2 µm. The PMUT membrane aspect ratio (k), i.e., the length-to-width ratio, determines the positions of higher resonant modes. Multifrequency (MF) arrays have been utilized to further improve the bandwidth of high frequency PMUTs for biomedical imaging applications [75]. A dual-frequency PMUT line array was proposed by Liu et al. [76]. The PMUT elements are designed with sputtered PZT of thickness 3 µm as the piezoelectric layer. The PMUT elements with diaphragm diameters 410 µm for 0.77 MHz and 230 µm for 2.30 MHz are implemented. The displacement sensitivities of 595 nm/V and 112 nm/V were reported for the PMUTs with 0.77 MHz and 2.3 MHz frequencies, respectively. Impedance measurements of the PMUT elements reported an EMCC of 4.3% and 5.6% for 0.77 MHz and 2.3 MHz, respectively. Line arrays for the two frequencies are arranged alternatively in rows and columns with rectangular grooves of width 100 µm between the arrays to limit interelement coupling effects. To further reduce the effects of interelement coupling, a misaligned ranking of the line arrays is implemented. The rectangular grooves etched in the silicon substrate and misaligned ranking of line arrays reduced the interelement coupling from 45% to 15%. Furthermore, a dual-frequency PMUT array was also proposed by Wang et al. [31], which utilized thin ceramic PZT of only 4 µm thickness. Due to the high piezoelectric coefficient of thin film ceramic PZT, the PMUT elements of the array have increased coupling efficiency of 6.3% and 5.7% for frequencies 1.2 MHz and 3.4 MHz, respectively. 

In developing high-performance PMUTs, various factors affect the transducer performance, including the piezoelectric material coefficient, electrode coverage, multielectrode, different device structures and resonant mode merging, large fill factor, and multifrequency operation. Table 2 outlines the state-of-the-art PMUTs and comparisons based on their achieved performance in terms of coupling efficiency, bandwidth, displacement, and acoustic sensitives. 

Several approaches in previous works have been reported to improve PMUT performance through achieving broad bandwidth, electromechanical coupling, and efficiency. Single PMUT designs use mode-merging techniques to achieve broad bandwidth. However, ultrasonic beam focus for better acoustic efficiency cannot be achieved through single PMUTs. PMUT arrays have also been developed to achieve broad bandwidth, and acoustic efficiency of the array is also enhanced through beam steering and beam forming [79,80]. Multifrequency PMUT arrays with single-frequency elements have been reported to enhance bandwidth [75,81,82], but using a single-frequency transducer element array involves inferior beam profiles [81]. Another approach in multifrequency PMUT arrays is to use elements of different sizes in the array. This improves the beam profiles and reduces cross coupling between elements [76]. However, using different element sizes in a PMUT array requires optimization of interelement spacing and pitch for achieving a high fill factor [81]. Fill factor is another important performance criterion for PMUT arrays. High fill factor results in better acoustic efficiency. PMUT arrays with cavity SOI fabrication and release holes have been utilized to improve the fill factor of PMUT arrays. Potential approaches for future advancements include the optimization of PMUT design for a desired application by defining figures of merit for expected performance. In medical imaging applications, high frequency and bandwidth are essential for better image resolution, whereas high acoustic efficiency is necessary for larger detection range. But high frequency signals have higher attenuation, limiting the imaging range/depth. Consequently, for imaging application, the figure of merit is the trade-off balance between broad bandwidth and acoustic efficiency, which can potentially be achieved by optimization of PMUT design parameters to enhance the coupling coefficient, array design for broader bandwidth, and fill factor improvement. Innovative device structures, exploring different piezoelectric material properties, novel creative fabrication techniques, and CMOS compatibility can further enhance PMUT performance.

MEMS devices need to be combined with electronic circuitry (IC) to execute signal processing and data transfer, minimize interconnections, and improve the signal-to-noise ratio (SNR) [37,83]. Shelton et al. [17] reported a CMOS-compatible PMUT with AlN as the piezoelectric layer [17]. AlN is a viable choice due to its low temperature thin film deposition compatible with the CMOS process, which enables monolithic integration of MEMS. An AlN-based PMUT on CMOS has been reported to implement a fully functional transceiver system with better performance and low package cost [35,37,73].

An AlN-based PMUT reported by Zamora et al. [84] utilizes MEMS on the CMOS platform from SilTerra foundry. This monolithic integration of PMUT with CMOS circuitry minimizes fabrication complexity, interconnections, and parasitic elements, improving the ultrasound system’s signal-to-noise ratio. Zamora et al. demonstrated a complete ultrasound system with a CMOS front-end transmitter and receiver using MEMS on the CMOS platform. The characterization of transmitter and receiver units of the ultrasound system was reported, and a comparison with the state-of-art systems revealed the competitive performance of the ultrasound system, with remarkable improvements in the electrical performance of the transmitter and noise performance of the receiver. Acoustic characterization of the PMUT array on CMOS was also demonstrated using a grating phantom 2.1 mm above the PMUT array in Flourinert FC-70 as the propagation medium. One row of the PMUT array was used for transmission, and another row for reception. A 2D image was reported, demonstrating the system’s capability for imaging applications.

Zamora et al. in [85] have demonstrated a single PMUT fully integrated and monolithically fabricated with the CMOS process. The PMUT reported is an AlN-based square structure with a side length of 80 µm and resonant frequency of 2.4 MHz in liquid medium. A pulse–echo investigation of the single PMUT on the CMOS ultrasound system was demonstrated, which reported 17.3 dB SNR, which is 27 dB higher than the non-integrated PMUTs.

## 6. PMUT Applications

PMUTs are implemented in ultrasound systems that demand miniaturization, low power consumption, cost-effective manufacturing, and integration with low-voltage CMOS circuitry. Further enhancements in design and fabrication have facilitated the use of PMUTs in several domains, including medical imaging, fingerprint sensing, wireless power transfer, and energy harvesting. Some of these PMUT applications are further explored in the following sections.

### 6.1. Medical Imaging

Ultrasound is widely implemented in imaging and medical diagnostic applications due to its non-invasive and non-destructive evaluation mechanism, portability, and low cost [86]. Due to their low input voltage and better CMOS compatibility compared to bulk transducers and CMUTs, PMUTs are employed in ultrasonic imaging and testing applications [13].

**Figure 18:**
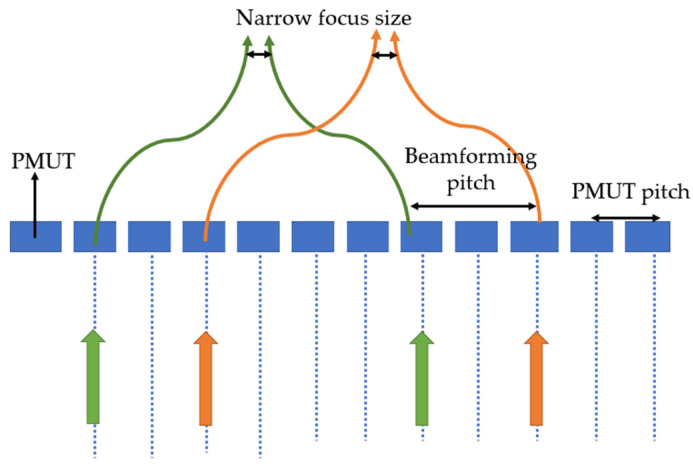
Beamforming using sub-array groups of 15 PMUT elements.

Pulse–echo imaging with high resolution requires a high fill factor to increase acoustic efficiency [13,69]. PMUTs based on cavity silicon-on-insulator (SOI) can be easily fabricated in high-fill-factor arrays [69]. An AlN-based PMUT was proposed by Lu et al. [79] for high-resolution short-range pulse–echo imaging [79]. FEM calculations and simulations demonstrated that a very high frequency of operation around 400 MHz is required to obtain a narrow acoustic beam (sub 100 µm). Therefore, instead of using a single PMUT element for high-frequency operation, an array of PMUT elements, with a working frequency of 8 MHz was proposed by Lu et al. The PMUT array utilized phased-array operation to achieve a narrow acoustic beam for high-resolution imaging. The reported PMUT array was fabricated on cavity SOI and consisted of 72 × 9 elements with an element diameter of 50 µm and interelement pitch of 70 µm. Ultrasound testing using needle hydrophone was demonstrated by Lu et al. using the beamforming technique with sub-array groups of 15 PMUT elements, as shown in Figure 18 [79]. It was reported that beamforming enhanced the acoustic pressure amplitude by three times when compared to the pressure amplitude from the same sub-array group of 15 elements. A 1.8–32 V charge pump application-specific integrated circuit (ASIC) interface was used to perform pulse–echo imaging experiments, which demonstrated the construction of B-mode images through a lateral scan of the phantom with a spatial resolution of 100 µm. The presented pulse–echo experimental results imply that the contrast ratio of images was enhanced through beamforming, while comparable echo signal amplitude was reported without beamforming.

Multifrequency imaging with a scandium-doped AlN PMUT array was proposed by Billen et al. [87]. The fabricated PMUT array consists of 32 × 32 PMUT elements with a pitch of 106 µm and a PMUT cell diameter of 60 µm to achieve a 7 MHz resonant frequency. A pulse–echo spectrum of the PMUT array was demonstrated for the 3–40 MHz frequency range, which reported amplitude peaks at 7 MHz and 20 MHz frequencies, viable for imaging within a 3 cm range. Acoustic pressure measurements of the PMUT array were demonstrated with a needle hydrophone in liquid with 10 V excitation amplitude of 7 MHz and 20 MHz frequency. The pressure amplitudes reported for the 7 MHz frequency excitation were 28.3 kPa at 18 mm and 3.7 kPa at 22 mm for 20 MHz excitation. Multifrequency imaging was demonstrated using nylon fibers in water for imaging. Images captured with 20 MHz actuation reported improved resolution compared to 7 MHz frequency, but the image size of nylon fibers was slightly smaller than the actual size due to low SNR. The SNR could be enhanced with a CMOS-compatible, scandium-doped AlN PMUT array, resulting in improved resolution and image quality [85].

In vitro and in vivo medical imaging using a 1D PMUT array of 64 elements was proposed by Savoia et al. [88] in which each element consisted of 184 circular PMUTs connected in parallel. Element-to-element spacing in the 1D array was reported to be 300 µm. The 1D PMUT array was integrated into an ultrasound probe using low-noise amplifiers to demonstrate small-signal acoustic characterization of the PMUT array. The PMUT was driven with a sine pulse of 5 V amplitude and 10 MHz frequency. The transmit and receive sensitivities of the PMUT were found as 31 kPa/V and 3.2 mV/kPa, respectively, with an 81% bandwidth. In vitro imaging investigation was demonstrated with the 1D PMUT array by connecting the ultrasound probe to a ULA scanner, which is a high-performance research scanner [89]. The ultrasound probe was excited at a 22 V sinusoidal voltage at 2 MHz for imaging a sectorial scan of a tissue-mimicking phantom. In vivo investigation was also demonstrated through a linear scan of carotid artery in a healthy human. These investigations demonstrated the promising potential of PMUT arrays for applications in in vivo and in vitro ultrasound imaging.

A 2D PMUT array proposed by Dausch et al. [28] consisted of 256 × 512 PMUT elements fabricated with through-silicon interconnects for integration with intracardiac catheters to perform in vivo, real-time 3D ultrasound imaging. Dausch et al. were the first to report the novel through-silicon interconnects in the PMUT array substrate, offering high-density arrays which can be integrated with Intracardiac Echo (ICE) catheter cabling. These through-silicon interconnects were implemented by vias which were reported to be etched using deep reactive ion etching (RIE) directly under the piezoelectric element from the back-side of the silicon substrate. In vivo imaging using a catheter-based PMUT array of 256 elements (64 × 4) connected to an ultrasound scanner was demonstrated on an adult swine. The PMUT array was excited by three-cycle sine wave pulses with 45 V peak-to-peak at 5 MHz frequency. B-mode images of the tricuspid, aortic, and pulmonary valves were captured. Dausch et al. were the first to report catheter-compatible cabling of PMUT arrays for in vivo, real-time, 3D intracardiac imaging.

Photoacoustic Imaging (PAI) is an emerging imaging technology which is based on the photoacoustic effect, i.e., the optoacoustic mission of sound waves due to absorption of light in a material [90]. Photoacoustic signals are wideband; therefore, it is essential to design the receiver transducer with broad bandwidth for better image quality and improved resolution [90]. A multifrequency PMUT array based on an AlN piezoelectric layer proposed by Cai et al. [91] consists of 72 elements arranged in 8 rows and 9 columns. The diameter of the PMUT elements gradually decreases from 300 µm to 180 µm in the row direction. The resonant frequency of the PMUT elements range from 1.6 MHz to 2.2 MHz. A pulse–echo experiment was carried out for the PMUT array in mineral oil at 1 V_pp_ drive voltage. A fractional bandwidth of 65% was achieved by the reported AlN-based multifrequency PMUT.

A thin ceramic PZT-based multifrequency PMUT array has been developed by Wang et al. [82] for endoscopic PAI. The PMUT array consists of 285 cells with resonant frequencies ranging from 1 MHz to 8 MHz. The PMUT elements have circular diaphragms with ceramic PZT of thickness 9 µm as the piezoelectric layer, which has a better piezoelectric coefficient than sputtered or sol-gel PZT or sputtered AlN [31]. However, the thinning and integration of ceramic PZT requires chemical mechanical polishing and wafer bonding fabrication techniques [31]. PAI imaging was performed using a laser source which generates short laser pulses of 720 nm wavelength at a 6 ns duration. Pencil leads 0.5 mm in diameter are used as imaging targets. The PMUT array is connected to a rotator to scan the target phantom. The output from the PMUT is amplified and processed to reconstruct photoacoustic images.

### 6.2. Acoustic and Mass-Based Sensing

PMUTs’ ability to attain higher operating frequencies [69], low-bias requirement [5], and inherent electromechanical coupling has enabled their implementation in acoustic and mass-based sensing applications. The PMUT as a mass-based sensor consists of a piezoelectric layer positioned between a top electrode, which is functionalized with a sensing layer, and a bottom electrode, and the entire structure is clamped at the edges [92]. The PMUT vibrates at its resonant frequency due to its piezoelectric property by applying an alternating voltage to the electrodes. When the PMUT sensor is exposed to an analyte gas or liquid, the sensing layer on the top electrode adsorbs the analyte molecules, causing mass loading on the PMUT and thereby shifting its resonant frequency. This shift in the device resonant frequency upon mass loading is utilized to determine the presence and concentration of the analyte molecules and is represented by Equation (14)

(14) $$\Delta f=-\frac{f_{0}}{2}\cdot \frac{\Delta m}{m}$$

Δf is the frequency shift, $f_{0}$ depicts the fundamental resonant frequency, m is the mass of the vibrating membrane, and Δm represents the change in mass upon mass loading. The sensing performance of PMUT-based mass sensors is characterized by the term sensitivity, which is defined as the ratio of the change in frequency caused due to the change in membrane mass upon analyte interaction. Equation (14) depicts that the fundamental resonant frequency of the PMUT is influenced by the design and material parameters, which affect the sensor sensitivity. Moreover, PMUT sensitivity is also affected by the sensing layer utilized in its operation [93].

**Figure 19:**
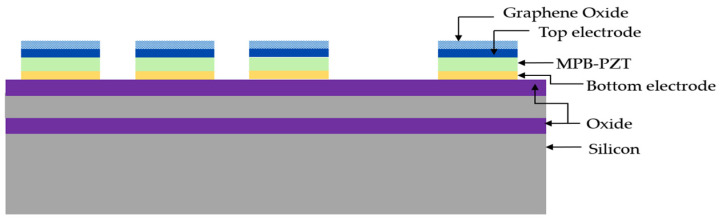
Schematic view of a PMUT array structure functionalized with a graphene oxide sensing layer.

Sun et al. have proposed and fabricated a high-sensitivity PMUT-array-based gas sensor for humidity detection [94]. In this work, a linear PMUT array consisting of 15 rectangular elements was individually functionalized with graphene oxide (GO) sheets that were responsible for analyte interaction. The structure of the rectangular PMUT sensor array comprised a 1.9 µm thick layer of morphotropic phase boundary composite lead zirconate titanate (MPB-PZT) as the piezoelectric active layer sandwiched between metal electrodes and placed on an SOI wafer. Figure 19 illustrates the PMUT array functionalized with GO sensing layers.

The microfabricated PMUT array was functionalized with GO thin film sheets using the facile drop-casting method and characterized with atomic force microscopy (AFM) to ensure uniform thickness. The authors reported an increase in the fundamental resonant frequency of the PMUT after functionalization with GO films due to a potential increase in membrane stiffness. The developed PMUT sensors demonstrated a humidity sensitivity of 719 Hz/%RH and relative sensitivity of 271.7 ppm/%RH when exposed to varying humidity levels in the range of 10–90% RH. The developed PMUT’s sensitivity was found to be notably higher in contrast with other high-frequency resonance-based humidity sensors [95,96].

**Figure 20:**
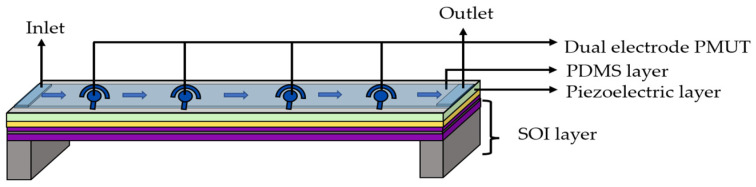
Schematic view of a PDMS-microchannel-integrated structure comprising dual-electrode PMUT elements.

PMUTs’ mass- and acoustic-sensing potential have further been extended to perform density sensing in the liquid phase for biomedical applications [97,98]. However, a notable limitation for acoustic density sensing was the requirement of a pair of transmitting and receiving electrodes to surround the analyte liquid, which further affected the design and fabrication process. To address this drawback, a single-cell PMUT in a dual-electrode configuration has been developed and analyzed for density sensing [99]. Roy et al. have further developed and investigated the sensing performance of a dual-electrode PMUT integrated with a microfluidic channel to identify the density variation in human blood [100]. The developed circular PMUT comprised PZT material of 500 nm thickness as the active piezoelectric layer along with gold-sensing and drive electrodes placed on a platinized SOI wafer, as illustrated in Figure 20. The device geometry included a PDMS microchannel comprising an inlet and outlet to allow for fluid movement. A linear array of three circular dual-electrode PMUTs was aligned and bonded to the PDMS channel to complete the sensor geometry.

The working mechanism of the dual-electrode PMUT is based on the mass loading effect, wherein a change in the PMUT’s resonant frequency is observed in response to the mass change induced by the fluid medium density. An alternating voltage is provided to the PMUT’s driving electrode while operating the device over the desired frequency range, and the maximum displacement of the membrane is recorded to determine the resonant frequency. Upon exposure to the analyte, the output voltage deduced from the sensing electrode is correlated to the shifted resonant frequency, which is used to identify and evaluate the analyte density. The developed PMUT sensor reported frequency shifts in the range of 20 kHz while exposed to fluid densities across a range of 774 kg/m^3^ to 1496 kg/m^3^ and attained a sensitivity of 26.3 Hz/(kg/m^3^).

### 6.3. Ultrasonic Fingerprint Sensing

Advancements in MEMS-based fabrication and CMOS integrability have enabled the use of array structures to attain a higher resolution in fingerprint sensing. The PMUT’s compact size [92], enhanced acoustic impedance matching, and ability to perform pulse–echo transmission while consuming less power have enabled its consistent use for ultrasonic sensing applications [41]. Further enhancements in piezoelectric materials [101] have facilitated the PMUT for CMOS integrability and, consequently, its use in real-time fingerprint sensing applications [102]. Horsley et al. have reported a monolithic ultrasonic sensor comprising PMUT array elements bonded to a CMOS wafer to facilitate commercial fingerprint sensing. The sensor geometry constituted an array size of 110 × 56 elements made of rectangular unimorph PMUTs comprising an area of 30 × 43 µm^2^. The PMUT elements were made up of an AlN piezoelectric active layer placed on a silicon substrate, bonded to a CMOS-based ASIC using Al/Ge anchors to provide electrical and mechanical connections [37].

The operating mechanism of the PMUT sensor involves pulse–echo recordings wherein an ultrasonic beam pulse is sent during the transmission (TX) phase and the echo is received during the receive (RX) phase. The CMOS wafer comprises the ASIC, which includes the transmission and reception circuitry. An alternating voltage of 24 V in 14 MHz pulses is provided to the columns of the PMUT arrays to form the pulse signal. Upon reception, the echo signal is demodulated and sampled through the receiver circuitry present in the CMOS wafer. The developed sensor attained an acoustic transmission pressure of 15 kPa and achieved a 75-micron lateral resolution and 150-micron axial resolution for an image of dimension 4.6 mm × 3.2 mm. The reported enhanced sensing performance enables the developed sensor for fingerprint-sensing applications based on real-time consumer standards.

### 6.4. Ultrasound Haptic Technology

Recent advancements in virtual reality, augmented reality, and robotics have ensured their integration in applications ranging from entertainment to providing effective training in surgery [103]. Haptic feedback is a component of these domains that give the user a feeling of touch and provides the user with sensory information on the amount of force applied. Commercially used acoustic transducers for haptics are too bulky to be integrated into portable products. Liu et al. have proposed using an array of 251 × 251 PZT PMUTs with a resonant frequency of 32.9 kHz to achieve ultrasonic haptic feedback due to their smaller size [104]. PZT was selected as the piezoelectric layer for its superior piezoelectric coefficient, generating higher acoustic pressure. The PMUTs were of 1000 μm radius and individually measured an output of 0.227 Pa of acoustic pressure when driven with 70 V peak-to-peak and 32.9 kHz sinusoidal bursts. Subsequently, focusing the ultrasound of the 251 × 251 array of PMUTs at 5 cm above the center of the array yielded a pressure level of almost 700 Pa at the focal point, sufficient to create a haptic sensation. These results demonstrate the potential of applying PMUTs in ultrasound haptic technology as a smaller, less power-consuming alternative to the traditional transducers.

### 6.5. Acoustic Power Transmission

The world’s growing focus on nanotechnology and implantable biomedical devices has garnered interest in technology to power them. Conventional portable power supplies such as batteries are not ideal for these applications due to their bulky size, fixed energy density, and limited lifespan [10]. Moreover, they carry associated risks such as heat release due to thermal events, toxic exposure due to electrolyte leakage, and device malfunction due to battery depletion. B. Herrera et al. presented a PMUT array for acoustic power transmission for intrabody powering applications [105]. A 70 × 70 element array was fabricated on a 300 m silicon wafer substrate. A 95 nm Ti bottom electrode was deposited, atop which 750 nm of AlN was reactively sputtered to form the piezoelectric layer. AlN was chosen over PZT for its higher biocompatibility, despite PZT having a higher electromechanical coupling coefficient. The array had a resonant frequency of 2 MHz in the dielectric oil testing medium. The results show a power output of 1 μW at 4 cm, which amounts to 7:1 μW/cm^2^ at a 77 mW/cm^2^ ultrasound intensity. This implementation achieves an output adequate for powering ASICs while staying below the FDA safety threshold of 720 mW/cm^2^.

### 6.6. Energy Harvesting

Dogheche et al. fabricated a PZT/Si PMUT for energy harvesting from inertial forces [106]. The diameter of the membranes of the fabricated PMUTs are 132 μm, 200 μm, and 400 μm, and the thickness of silicon and PZT are set to 1 and 2 μm, respectively. The mechanical excitation source from which the energy is harvested is weak inertial energy in the form of handshakes with an acceleration between 0.5 g and 2 g (g, gravitational acceleration = 9.8 ms^−2^). The results demonstrate two mechanical behaviors: linear (elastic) and non-linear (bistable). In the elastic mode, the voltage levels vary with the level of mechanical excitation due to membrane deformation. The bistable behavior was observed by two sharp, equal, and opposite response signals corresponding to the stress inversion (compression to extension and extension to compression), both with their respective flow of generated electrical charges. The voltage response recorded on a 1 MΩ input impedance oscilloscope shows an induced voltage of up to 15 mV, which implies the utility of PMUT-based energy harvesters for powering biomedical implants in the human body.

### 6.7. Particle Manipulation

The need to independently manipulate microscale objects in a non-contact way has been extensively researched for its uses in biology to ensure the object’s integrity without disrupting any internal processes. Cheng et al. have fabricated PZT-based PMUT arrays with a diaphragm diameter of 60 μm and a resonant frequency measured at approximately 8 MHz [107]. A 1D array consisting of 20 elements was fabricated for particle manipulation with a Pin Grid Array (PGA) cavity filled with 4 μm SiO_2_ beads in a distilled water medium. Particle manipulation was achieved by selecting the order of excitation of the PMUTs, enabling the beads to move from element to element in the 1D array. This was observed due to the generation of an acoustic gradient in the array, which facilitates the movement of the beads from a high acoustic potential to a lower one. Frequency variation of the PMUTs demonstrated various patterns in the bead clusters. This presents PMUTs potential in precisely manipulating particles and controlling the number of particles in a cluster.

## 7. Conclusions and Perspectives

PMUTs have drawn increased attention in recent years due to their low power consumption and potential monolithic CMOS integration. PMUTs offer unique advantages over conventional bulk piezoelectric transducers and CMUTs mainly due to lesser geometric constraints, microfabrication, and low power consumption. Considerable progress has been made in previous works to improve the performance of PMUTs, with keen focus on enhancing transducer properties, namely resonant frequency, EMCC, bandwidth, and acoustic pressure output. The resonant frequency in PMUTs is dependent on material properties, membrane shape, and size, consequently offering better design flexibility for high frequency applications. The EMCC is enhanced by electrode size optimization with analytical equivalent circuit models of PMUTs. Three-dimensional membrane structures have been utilized in past research which significantly improve the electromechanical performance of PMUTs. Innovative piezoelectric materials, including thin ceramic PZT, monocrystalline PZT, and scandium-doped AlN with high piezoelectric coefficients have been reported in recent work on PMUTs to improve the electromechanical coupling efficiency. In ultrasonic imaging applications, the bandwidth of the transducer is crucial in achieving better image resolution. PMUTs with exclusive membrane structures, such as ribbon-shaped (rectangular membranes with large aspect ratios) and ring-shaped membranes, have been utilized to broaden the bandwidth through merging two or more resonant modes. PMUTs with backing layers have also been described which double bandwidth performance. The acoustic efficiency in PMUTs is improved by partially clamped membrane boundaries, curved/dome-shaped membrane structures, and the beamforming approach. The implementation of venting rings and release holes on PMUT arrays has also demonstrated improved acoustic output in PMUTs.

PMUT arrays with different element sizes have been reported to enhance the bandwidth and acoustic efficiency of PMUTs. For PMUT arrays, the fill factor of the array design is an important factor for achieving better acoustic efficiency. PMUTs fabricated with cavity SOI can be designed with smaller dimensions and interelement spacing, resulting in a high fill factor. PMUT array designs with reusable shared etch holes have also been reported to improve the fill factor significantly. Interelement cross-coupling issues for PMUT arrays have also been addressed in previous works to improve displacement sensitivity and PMUT performance through designing etched grooves between PMUT arrays. Multifrequency PMUT arrays have been reported to further enhance the bandwidth of PMUTs for their applications in high resolution ultrasonic and photoacoustic imaging. The monolithic CMOS integration of PMUTs facilitates high fabrication yield in developing miniaturized ultrasound systems with improved transmit/receive performance and increased SNR. PMUTs have a broad range of applications in medical imaging, mass-based sensing, fingerprint sensing, acoustic power transmission, and promising potential in energy harvesting, haptic feedback technology, and particle manipulation. Potential advancements in PMUT design for a desired application involves an optimization approach that considers the interlinked transduction metrics and inherently coupled design parameters [51,108,109].

Future advancements in PMUT technology will focus on the development of innovative device structures, fabrication techniques, piezoelectric materials, and improved analytical modeling. Further optimization of PMUT on CMOS for better integration compatibility may facilitate PMUTs to emerge as a prominent next-generation ultrasound technology for applications in actuation, sensing, and imaging.

## Figures and Tables

**Table 1:** Commonly used piezoelectric materials in PMUTs and their properties.

Property	Units	PZT [26]	AlN [38]	ZnO [39]
Density	Kg/cm^3^	7500	3260	5680
Young’s Modulus	GPa	127	410	209
Piezoelectric Coefficient e_31_	C/m^2^	−8 to −12	−1.05	−1.0
Converse Piezoelectric Coefficient d_33_	pm/V	60–130	3.9	5.9

**Table 2:** Comparison of recently reported PMUTs and their attained performances.

PMUT	Element Size (µm)	Operating Frequency (MHz)	Displacement Sensitivity (nm/V)	Pressure Sensitivity (kPa/V)	Electromechanical Coupling	Bandwidth	Fill Factor
Dome Shaped PMUT array, Niobium doped (13%) PZT [57]	74 to 94	5	--	85	45%	55%	57 domes with pitch 400 µm
50 × 50 PMUT array, AlN [72]	48	12.62	3.25	--	1.6%	--	67%, pitch 52 µm
15 × 9 PMUT array, PZT [60]	40	10	--	450	12.5%	--	High, pitch 60 µm
Annular PMUT array with 1261 cells, AlN [69]	25	18.6	2.5	9	--	4.9 MHz	High, pitch 30 µm, 1061 cells/mm^2^
24 × 8 PMUT array, AlN [77]	50	22	1.8	14	--	11.5	17%, pitch 100
128 + 128 PMUT array, Thin PZT ceramic [31]	220, 120	1.2, 3.4	110, 30	--	6.3%, 5.7%	0.84 MHz—54%, 2.4 MHz—17%	Pitch 380 µm
120 + 192 PMUT array Sputtered PZT [76]	410, 230	0.77, 2.3	595, 112	53, 73	4.3%, 5.6%	--	--
Square PMUT, AlN [35]	80	1.5	--	4.9	1.14%	--	--
Curved PMUT, AlN [55]	120 membrane diameter, 550 radius of curvature	3.86	45	--	2.1%	--	Low
Zero-Bending Square PMUT [78]	200	2.21	123	--	0.406%	--	--
